# Reliability and validity study of the “5Cs” hesitancy scale for maternal influenza vaccination among pregnant and postpartum women

**DOI:** 10.1186/s40249-025-01295-8

**Published:** 2025-04-30

**Authors:** Fanyu Zeng, Bingcheng Du, Hong Jiang, Min Zheng, Xiu Qiu, Fen Li, Nianhua Yi, Yinglan Wu, Yuanying Ma, Changhui Li, Chunyi Gu, Lei Wang, Fengyun Yang, Longmei Jin, Yanran Yang, Xu Qian

**Affiliations:** 1 School of Public Health, Key Laboratory of Health Technology Assessment, National Health Commission, Key Laboratory of Public Health Safety, Ministry of Education, Fudan University, Shanghai, 200032 China; 2 Department of Statistics, University of Toronto, Toronto, Canada; 3 Yunnan Maternal and Child Health Care Hospital, Kunming, 650021 China; 4 Guangzhou Women and Children’s Medical Center, Guangzhou, 510623 China; 5 The First Affiliated Hospital of Xi’an Jiao Tong University, Xi’an, 710061 China; 6 Department of Maternal Health Care, Huazhong University of Science and Technology Tongji Medical College Maternal and Child Health Hospital of Hubei Province, Wuhan, 430070 China; 7 Hunan Provincial Maternal and Child Health Care Hospital, Changsha, 410008 China; 8 Women’s Hospital School of Medicine, Zhejiang University, Hangzhou, 310006 China; 9 Urumqi Youai Hospital, Urumqi, 830001 China; 10 Liaoning Provincial Maternal and Child Health Hospital, Shenyang, 110005 China; 11 Obstetrics and Gynecology Hospital, Fudan University, Shanghai, 200011 China; 12 Shanghai Jiading District Maternal and Child Health Care Hospital, Shanghai, 201800 China; 13 Shanghai Minhang Maternal and Child Health Care Hospital, Shanghai, 201102 China; 14 Duke Kunshan University, Kunshan, 215316 China

**Keywords:** Influenza vaccine, Vaccine hesitancy, Pregnancy, Childbirth, Reliability, Validity

## Abstract

**Background:**

Maternal influenza vaccine hesitancy plays a vital role in the low rates of vaccination. However, instruments to appropriately assess perinatal influenza vaccine hesitancy are unavailable. This study aimed to develop the Maternal Influenza Vaccine Hesitancy Scale based on the 5C vaccination hesitancy scale, containing the subscales of confidence, complacency, constraints, calculative, and collective responsibility, and to provide a preliminary overview of the current hesitancy on maternal influenza vaccination in China.

**Methods:**

A cross-sectional survey, from January to March 2024, was carried out among 2035 pregnant and postpartum women from nine provincial-level administrative divisions representing eastern, central, western, and northeastern areas of China. Reliability was evaluated by internal consistency reliability and split-half reliability, and a Cronbach’s alpha coefficient > 0.7 was considered acceptable. Construct validity was assessed using confirmatory factor analysis (CFA), with good model fit defined as root mean square error of approximation (RMSEA) < 0.100, normed fit index (NFI) > 0.9, comparative fit index (CFI) > 0.9, and Tucker-Lewis index (TLI) > 0.9.

**Results:**

Confirmatory factor analysis results supported the five-factor structure of the scale (RMESA = 0.098, CFI = 0.921, TLI = 0.903, NFI = 0.918). The Cronbach's alpha coefficients for the scale as well as the subscales ranged from 0.802 to 0.958. Among five subscales, collective responsibility (2.73 ± 0.63) scored highest, while complacency (2.16 ± 0.69) and constraints (2.17 ± 0.69) were the lowest.

**Conclusions:**

The Maternal Influenza Vaccine Hesitancy Scale developed in this study is a reliable and valid instrument to measure the influenza vaccine hesitancy of pregnant and postpartum women. It is recommended that interventions including health education and improving the access to the vaccination service be carried out to reduce the maternal influenza vaccination hesitancy.

**Graphical Abstract:**

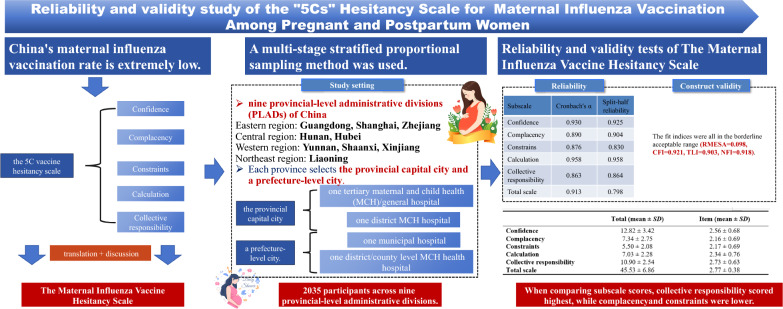

**Supplementary Information:**

The online version contains supplementary material available at 10.1186/s40249-025-01295-8.

## Background

Pregnant women are particularly susceptible to influenza [1]. In 2017, a working group convened by the World Health Organization (WHO) estimated symptomatic laboratory-confirmed influenza (LCI) ranged from 0.10 to 486 per 10,000 pregnant women [2]. During 2009–2010, a total of 81 reported cases of the H1N1 pandemic in 2009 occurred among pregnant women in Beijing, included 25 (30.9%) severe cases and 6 (6.2%) death cases [3]. An active surveillance among pregnant women in Suzhou, China, found that influenza incidence increased from 0.7/100 person-months in 2016 to 2.1/100 person-months in 2018 [4]. Further, influenza infection was reported significantly associated with the increased risk of adverse pregnancy and birth outcomes [5]. A systematic review consisting of eight retrospective cohort studies and two case–control studies showed that mothers with H1N1 illness had a higher rate of stillbirth ([6]. A meta-analysis involving 29,542 congenital anomalies cases found first trimester maternal influenza was associated with an increased risk of congenital anomaly, such as neural tube defects, and hydrocephaly [7].

Maternal immunization is a safe and effective strategy to protect the health of the mother and their fetus/baby. Systematic reviews and empirical studies have shown that influenza vaccinations during pregnancy have not shown a significantly increased risk of adverse outcomes among pregnant women and their offspring [8–12]. Studies related to the vaccine efficacy/effectiveness showed that influenza vaccination during pregnancy protected women from influenza infection [10, 13–15]. More importantly, maternal influenza vaccination offers the advantage of endowing the fetus with maternal antibodies against influenza prior to delivery [16]. Nevertheless, the rate of maternal influenza vaccination remains unsatisfied worldwide. In the United Kingdom and the United States, 43.7% and 61.2% pregnant women received influenza vaccination from 2019 to 2020, lower than the national target of 75% [17, 18]. In China, this rate was extremely low as only 0.91% of pregnant women in Shenzhen were vaccinated in 2016, and this increased slightly to 1.88 in 2019 [19, 20]).

Low rate of vaccination is highly correlated with high level of perceived vaccine hesitancy. It is important to assess and understand the current situation of maternal influenza vaccine hesitancy. In 2012, the Strategic Advisory Group of Experts on Immunization (SAGE) established a working group to create a model to explain the determinants of vaccine hesitancy. The 3C model, containing the subscales of confidence, complacency and convenience, was established to understand vaccine hesitancy globally. Confidence is defined as trust in the vaccines, the system that delivers them, and the motivations of policy-makers [21]. Complacency indicates a low perceived risk of vaccine-preventable diseases [21]. Convenience measures the degree of an individual’s ability to access vaccines [21]. In 2018, based on the 3C model, Betsch et al. established the 5C model of vaccine hesitancy by adding two dimensions—‘calculation’ and ‘collective responsibility’. Individuals having the characteristics of calculation are assumed to engage more in information searching and be more hesitant towards vaccines, due to the large amount of vaccine misinformation. Collective responsibility considers an individual’s willingness to protect others through herd immunity.

So far, little is known about the level of vaccine hesitancy on influenza vaccination among pregnant women due to the lack of valid tools for suitable assessment. Therefore, we proposed this study to construct a maternal influenza vaccination hesitancy scale based on the 5C vaccination hesitancy scale, to carry out a validity and reliability analysis, and to provide a preliminary description of the current situation of maternal influenza vaccination hesitancy in China.

## Methods

### The adaptation of 5C scale

The Maternal Influenza Vaccine Hesitancy Scale was developed by Betsch et al. based on the 5C scale assessing influenza vaccination hesitancy among the general population in 2018. The original 5C scale included 15 items with five subscales: confidence (three items), complacency (three items), constraints (three items), calculative (three items), collective responsibility (three items). When adapted for usage in maternal influenza vaccine hesitancy, the structure of the scale remains unchanged, containing the same five subscales. We added “influenza” before vaccine/vaccination and the specific time “during pregnancy/postpartum” in each item. Furthermore, items related to the safety and effectiveness of fetuses/babies’ health were added in each confidence, complacency and collective responsibility subscale. In the confidence subscale, two additional items were added: (a)“ I am completely confident that influenza vaccination during pregnancy/postpartum is safe for the fetus/baby with breastfeeding.”, (b)“ Influenza vaccination during pregnancy/postpartum is effective for the fetus/baby with breastfeeding.”. In the complacency subscale, one additional item was added: “The fetus/baby’s immune system is so strong; it protects them against influenza.”. In the collective responsibility subscale, one additional item was added: “Getting the influenza vaccination during pregnancy/postpartum is important because it helps protect the fetus/baby.” As a result, a total of 19 items were established for the Maternal Influenza Vaccine Hesitancy Scale (Table 2).

**Table 1:** Individual characteristics of pregnant and postpartum women (n = 2035)

Characteristics	*n* (%)	Characteristics	*n* (%)
Number of participants	2035 (100)	Annual household income (USD 1000)^*^	
Age (years, mean ± SD)	30.3 ± 4.18	< 6.86	469 (23.0)
Age (years)		6.86–27.45	1171 (57.6)
< 20	11 (0.5)	> 27.45	395 (19.4)
20–35	1694 (83.2)	Marital status	
≥ 35	330 (16.3)	Married	1977 (97.1)
Perinatal stage		Divorced/widowed/remarried	58 (2.9)
First trimester	495 (24.3)	Family structure	
Second trimester	506 (24.8)	Nuclear family	1139 (56.0)
Third trimester	519 (25.6)	Stem family	631 (31.0)
Postpartum period	515 (25.3)	Extended family	202 (9.9)
Ethnic group		Others	63 (3.1)
Han ethnic group	1859 (91.4)	Intention to/ being breastfeeding	
Others	176 (8.6)	Yes	1847 (90.8)
Residence		No	188 (9.2)
Urban	1633 (80.2)	Parity	
Rural	402 (19.8)	Primiparous	1133 (55.7)
Educational level		Multiparous	902 (44.3)
Junior school or below	203 (10.0)		
Senior high school/vocational school	257 (12.6)		
Junior college	493 (24.2)		
College and above	1082 (53.2)		

^*^The exchange rate between USD and CNY is 7.29

*SD* Standard deviation

Prior to the survey, the initial translation was completed by a native Chinese researcher, FYZ. Two senior experts, HJ and XQ, with more than 20 years of research experience in maternal and child health care evaluated and revised translation to ensure the accuracy and cultural appropriateness.

### Study setting

From 17 January 2024 to 14 March 2024, the survey, using multi-stage stratified proportional sampling method, was conducted in nine provincial-level administrative divisions (PLADs) of China, including Guangdong, Zhejiang and Shanghai, representing eastern China; Hunan and Hubei, representing central China; Yunnan, Shaanxi and Xinjiang, representing western China; and Liaoning representing northeastern areas in China. The sample size in each PLAD was allocated based on its proportion of the total deliveries in the nine PLADs in 2022. In each PLAD, four sites were selected, one tertiary maternal and child health (MCH)/general hospital and one district MCH hospital in the provincial capital, as well as one municipal hospital and one district/county level MCH hospital in a prefecture city. The sample size in each city was allocated based on the proportion of deliveries in the city relative to the total number of deliveries in the PLAD in 2022. Furthermore, the sample size of each medical institution in the same city was allocated based on the ratio of delivery numbers of the two institutions in 2022.

### Participants and data collection

We recruited pregnant women and postpartum women who scheduled antenatal health examinations during the survey period. The inclusion criteria included: (1) the participants were aged 18 and above; (2) the participants were either pregnant or within four months postpartum, and (3) the participants were able to understand and fill in the questionnaire by themselves.

The research has obtained the ethical approval of School of Public Health, Fudan University (IRB#2023-12-1093). During the survey, the trained staff invited eligible pregnant and postpartum women to participate in the survey and provided participants with the electronic QR code of a self-administered questionnaire via Wenjuanxing (www.wjx.cn), a widely used online questionnaire survey platform. Pregnant women invited to participate in the anonymous survey were required to scan the QR code via a mobile phone or other digital devices. At the beginning of the survey, an online informed consent process was set with a brief introduction, including the aim and contents of the online questionnaire and the estimated time of completion (7.18–32.60 min). All Participants provided informed consents. They could decide whether to continue or withdraw from the survey at any time during the survey. The online questionnaire included sociodemographic characteristics of participants and the Maternal Influenza Vaccine Hesitancy Scale, etc.

Quality control measures are as follows: (1) One quality control question was included in the questionnaire. If a respondent answered this question incorrectly, the questionnaire was considered invalid. (2) Pilot survey was conducted before the launch of the study, and it took at least 5 minutes to complete. Any questionnaire submitted in less than 3 minutes was deemed invalid.; (3) Questionnaires containing the same answer for every question were treated as invalid.

### Questionnaires and measurements

#### Socio-demographic characteristics

During the survey, we collected the following socio-demographic information from the participants: age, perinatal stage, ethnic group, residence, educational level, annual household income, marital status, family structure, breastfeeding intention for pregnant women or breastfeeding status for postpartum women, parity.

#### The maternal influenza vaccine hesitancy scale

The Maternal Influenza Vaccine Hesitancy Scale consists of 5 items in the confidence subscale, 4 items in both the complacency and collective responsibility subscales, 3 items in the constraints and calculation subscale, with 19 items in total. The 4-point Likert scale, with scores ranging from 1 (Strongly disagree) to 4 (Strongly agree) was used for each item. High levels of confidence and collective responsibility indicated low levels of vaccine hesitancy [22]. High levels of complacency, constraints and calculation represented high levels of vaccine hesitancy [21, 22]. Therefore, items were reversely coded for the 4-point Likert in the complacency, constraints and calculation subscale. A mean score of items under each subscale was computed. Higher scores in each subscale indicated that pregnant women had higher confidence in the influenza vaccine, lower complacency about the risks of influenza, fewer constraints in accessing influenza vaccination, a more calculative approach to gathering influenza vaccine-related information, and a stronger sense of collective responsibility to protect others through influenza vaccination.

### Data analysis

#### Reliability

The online questionnaire results were exported and sorted in Microsoft Excel 16.0 (Microsoft Corporation, Redmond, USA), and analyzed by SPSS 25.0 (IBM Corporation, Chicago, USA). Reliability analysis included internal consistency reliability and split-half reliability. We used Cronbach’s α coefficient to evaluate the internal consistency reliability and the Spearman-Brown coefficient to evaluate the split-half reliability between odd questions and even questions. A reliability coefficient above 0.7 was acceptable for the whole scale [23].

#### Validity

Validity was analyzed using construct validity. AMOS 23.0 (IBM Corporation, Chicago, USA) was used to analyze the construct validity. The construct validity of the questionnaire was evaluated by confirmatory factor analysis (CFA). Maximum likelihood estimation was used in CFA analysis. The good model fit for CFA was defined as: (1) root mean square error of approximation (RMSEA) < 0.1; (2) normed fit index (NFI) > 0.9; (3) comparative fit index (CFI) > 0.9; (4) Tucker-Lewis index(TLI) > 0.9[24].

## Results

### Participants’ characteristics

A total of 2188 questionnaires were collected, of which 2035 were valid (Table 1). The average age of the respondents was 30 years old, ranging from 18 to 48. The participants were evenly distributed in the 1st, 2nd, 3rd trimester of pregnancy and postpartum, with each stage accounting for over 20%. More than 90% (1859, 91.4%) were Han Ethnic. Additionally, 80% (1633, 80.2%) were urban residents. Most of the pregnant women (1368, 67.2%) had attained a university/college level and above education. More than half (1171, 57.5%) of the participants had an annual household income between 6850 and 27,400 USD (USD∶CNY = 1∶7.29). Nearly ninety percent of the participants intended to breastfeed (1847, 90.8%). Additionally, almost fifty percent of the respondents were primiparas (1134, 55.7%).

### Construct validity

A confirmatory factor analysis was conducted. The fit indices were all in the acceptable range (RMESA = 0.098, CFI = 0.921, TLI = 0.903, NFI = 0.918).

### Reliability

For the Maternal Influenza Vaccine Hesitancy Scale, the total Cronbach’s α coefficient was 0.915, ranging from 0.802 to 0.958 for five subscales (Table 2). The split-half coefficients were 0.793 for the total scale and from 0.722 to 0.904 for five different subscales.

**Table 2:** Reliability of the maternal influenza vaccine hesitancy scale (*n* = 2035)

Item	Cronbach’s α	Split-half reliability
Confidence	0.930	0.871
1. I am completely confident that influenza vaccine during pregnancy/postpartum are safe for me.		
2. I am completely confident that influenza vaccine during pregnancy/postpartum are safe for the fetus/baby with breastfeeding.		
3. Influenza vaccination during pregnancy/postpartum is effective for me.		
4. Influenza vaccination during pregnancy/postpartum is effective for the fetus/baby with breastfeeding.		
5. Regarding influenza vaccines during pregnancy/postpartum, I am confident that public health authorities decide in the best interest of the community.		
Complacency	0.890	0.904
6. Influenza vaccination is unnecessary because the influenza is no longer common during pregnancy/postpartum.		
7. My immune system is so strong; it protects me against influenza during pregnancy/postpartum.		
8. The fetus/baby’s immune system is so strong; it protects them against influenza.		
9. Influenza is not severe enough that I should get vaccinated during pregnancy/postpartum.		
Constraints	0.876	0.722
10. Everyday stress during pregnancy/postpartum prevents me from getting influenza vaccination.		
11. For me, it is inconvenient to receive influenza vaccination during pregnancy/postpartum.		
12. Visiting the doctors makes me feel uncomfortable, this keeps me from getting the influenza vaccination during pregnancy/postpartum.		
Calculation	0.958	0.857
13. When I think about getting influenza vaccination during pregnancy/postpartum, I weigh its benefits and risks to make the best decision possible.		
14. For influenza vaccination during/after each pregnancy, I closely consider whether it is useful for me.		
15. It is important for me to fully understand the topic of influenza vaccination before I decide to get vaccinated during pregnancy/postpartum.		
Collective responsibility	0.802	0.770
16. When everyone is vaccinated, I don’t have to get influenza vaccination during pregnancy/postpartum.		
17. I get influenza vaccination during pregnancy/postpartum because I can also protect those with weaker immune systems.		
18. Getting the influenza vaccination during pregnancy/postpartum is important because it helps protect the fetus/baby.		
19. Influenza vaccination is a collective effort to prevent the spread of diseases during pregnancy/postpartum.		
Total scale	0.915	0.793

### The status of influenza vaccination hesitancy among Chinese pregnant and postpartum women

The average total score of the scale was 45.53 ± 6.96 (full score 76), with the highest average scores of 2.73 ± 0.63 in the collective responsibility items (full score 16), while the subscales of complacency and constraints were relatively lower, scored 2.16 ± 0.69 (full score 16) and 2.17 ± 0.69, respectively (full score 12) (Table 3).

**Table 3:** The status of influenza vaccination hesitancy among Chinese pregnant and postpartum women (*n* = 2035)

	Total (mean ± *SD*)	Item (mean ± *SD*)
Confidence	12.82 ± 3.42	2.56 ± 0.68
Complacency	7.34 ± 2.75	2.16 ± 0.69
Constraints	5.50 ± 2.08	2.17 ± 0.69
Calculation	7.03 ± 2.28	2.34 ± 0.76
Collective responsibility	10.90 ± 2.54	2.73 ± 0.63
Total scale	45.53 ± 6.86	2.77 ± 0.38

*SD* Standard deviation

Among the nine PLADs surveyed, Shanghai achieved the highest average total score on the scale (46.68 ± 5.855), while Guangdong recorded the lowest (44.87 ± 6.316) (Supplementary Table 1).

## Discussion

In this study, we adapted the original 5C scales to a Chinese context and added four items to address the concerns of pregnant women and their families regarding the safety and effectiveness of influenza vaccines during pregnancy and postpartum period. The construct validity of the Maternal Influenza Vaccine Hesitancy Scale was evaluated by CFA which revealed a robust five-factor structure for the scale, including confidence, complacency, convenience, calculation, and collective responsibility subscales. For CFA, we chose some modeling fit indices, such as RMESA and CFI, which were less affected by the large sample size [25]. The Cronbach’s α coefficient of the Maternal Influenza Vaccine Hesitancy Scale, both for the total scale and each subscale, were all similar or higher than those of the original 5C scale [22]. By use of the scale, the status of influenza vaccine hesitancy among pregnant and postpartum women could be appropriately measured, which could provide evidence for developing influenza vaccination intervention strategies for target population.

Our findings indicated that the status of influenza vaccine hesitancy among Chinese pregnant and postpartum women. Ccompared to other subscales, the scores of complacency and constraints were relatively low. This suggests a low level of perceived risk of influenza infection during pregnancy and limited access to influenza vaccination for individuals. Consistent with previous studies [26–29], perceiving a lower personal risk of contracting the disease was found to increase influenza vaccine hesitancy during pregnancy. In a systematic review about maternal influenza vaccination decision-making [30], pregnant women who felt that they were susceptible to influenza had almost two-times higher odds of vaccination than those who did not feel at risk. Furthermore, pregnant women who believed that influenza could harm their baby were four times more likely to be vaccinated than those who did not [31–33]. Additionally, previous studies suggested pregnant women with more convenient maternal influenza vaccination service had a threefold higher probability of vaccination compared to those with limited assess [34, 35]. Addressing the low access of vaccination service has the large potential for uptaking vaccination service.

The strengths of our study included that we evaluated the validity and reliability of the Maternal Influenza Vaccine Hesitancy Scale across eastern (Guangdong, Zhejiang, Shanghai), central (Hunan and Hubei), western (Yunnan, Shaanxi and Xinjiang) and northeastern (Liaoning) regions of China, covering a large part of the country and improved representativeness of the study . Additionally, the proportion of ethnic minorities in our study population aligned with the findings of the 7th National Population Census of China in 2020 [36]. The instrument filled in the gap of comprehensive assessment of the influenza vaccine hesitancy among pregnant and postpartum women. The satisfactory reliability and validity of the scale demonstrated by our study suggested that the instrument could be used for assessing maternal influenza vaccine hesitancy in China and potentially for other similar countries/regions in the world. Moreover, the instrument could be used to understand the determinants of maternal influenza vaccine hesitancy.

The limitations of this study lie in the fact that the survey was conducted exclusively during the influenza season, which may under-estimate influenza maternal vaccine hesitancy.In addition, the reliability and validity study were only conducted in China. It is advocated to carry out further tests among pregnant and postpartum women in different countries and regions.

## Conclusions

The Maternal Influenza Vaccine Hesitancy Scale has good reliability and validity and can be used to assess maternal influenza vaccine hesitancy in China. The preliminary survey showed low levels on the complacency and constraints on the vaccination, suggesting that maternal perceptions on influenza risk and benefits of influenza vaccination, and the constraints of influenza vaccination service among pregnant and postpartum women need to be improved in China. It is suggested that interventions including health education and improving the access of the vaccination service be carried out to reduce the maternal influenza vaccination hesitancy.

## Supplementary Information

Supplementary Material 1.

## Data Availability

The datasets used and analyzed during the current study are available from the corresponding author (h_jiang@fudan.edu.cn) upon reasonable request.

## Abbreviations

WHO: World Health Organization
LCI: Laboratory-confirmed influenza
SAGE: The strategic advisory group of experts on immunization
PLADs: Provincial-level administrative divisions
MCH: Maternal and child health
CFA: Confirmatory factor analysis
RMSEA: Root mean square error of approximation
NFI: Normed fit index
CFI: Comparative fit index
TLI: Tucker-Lewis index
