# Supplementary material for: Reliability and validity study of the “5Cs” hesitancy scale for maternal influenza vaccination among pregnant and postpartum women
Source: Infect Dis Poverty. 2025 Apr 30;14:34. doi: 10.1186/s40249-025-01295-8 (PMC12042492; doi:10.1186/s40249-025-01295-8)
Supplement: Supplementary file 1 — Supplementary Material 1. [file 40249_2025_1295_MOESM1_ESM.docx]

**Supplementary Table 1. Results of** **preliminary application (n=2035)**

|  | **Confidence**  **(mean±SD)** | **Complacency**  **(mean±SD)** | **Constraints**  **(mean±SD)** | **Calculation**  **(mean±SD)** | **Collective Responsibility**  **(mean±SD)** | **Total scale**  **(mean±SD)** |
| --- | --- | --- | --- | --- | --- | --- |
| **Guangdong** | 13.06±3.161 | 6.96±2.739 | 5.16±2.038 | 8.7±2.129 | 10.99±2.442 | 44.87±6.316 |
| **Zhejiang** | 13.08±3.364 | 7.3±2.643 | 5.39±2.072 | 9.2±2.133 | 11.09±2.278 | 46.06±6.892 |
| **Shanghai** | 12.99±2.848 | 7.22±2.459 | 5.33±1.658 | 9.55±1.851 | 11.6±1.892 | 46.68±5.855 |
| **Hunan** | 12.79±3.345 | 7.33±2.56 | 5.64±2.099 | 8.9±2.294 | 10.96±2.545 | 45.62±7.041 |
| **Hubei** | 12.58±3.661 | 7.54±2.822 | 5.43±2.244 | 8.94±2.415 | 10.77±2.634 | 45.26±7.264 |
| **Yunnan** | 12.89±3.787 | 7.61±2.949 | 5.57±2.235 | 8.86±2.358 | 10.69±2.793 | 45.62±7.497 |
| **Shaanxi** | 11.96±3.179 | 7.65±2.698 | 5.92±1.92 | 9.03±2.472 | 10.7±2.576 | 45.26±7.052 |
| **Xinjiang** | 13.21±3.858 | 7.2±3.329 | 5.55±2.386 | 8.63±2.551 | 10.8±2.996 | 45.38±7.731 |
| **Liaoning** | 12.74±3.253 | 7.35±2.421 | 5.57±1.855 | 9.04±2.13 | 10.8±2.364 | 45.51±6.857 |
| **Total**  **(mean±SD)** | 12.82±3.42 | 7.34±2.75 | 5.50±2.08 | 8.96±2.28 | 10.90±2.54 | 45.53±6.86 |
| **Item**  **(mean±SD)** | 2.56±0.68 | 2.16±0.69 | 2.17±0.69 | 2.99±0.76 | 2.73±0.63 | 2.77±0.38 |
